# Epigallocatechin gallate (EGCG) alleviates vascular dysfunction in angiotensin II-infused hypertensive mice by modulating oxidative stress and eNOS

**DOI:** 10.1038/s41598-022-21107-5

**Published:** 2022-10-21

**Authors:** Nurul Aiza Mohd Sabri, Siew-Keah Lee, Dharmani Devi Murugan, Wei Chih Ling

**Affiliations:** 1 Department of Pre-Clinical Sciences, Faculty of Medicine and Health Sciences, Universiti Tunku Abdul Rahman, 43000 Kajang, Selangor Malaysia; 2 Department of Pharmacology, Faculty of Medicine, Universiti Malaya, 50603 Kuala Lumpur, Malaysia

**Keywords:** Pharmacology, Cardiovascular biology

## Abstract

Epigallocatechin gallate (EGCG) has been shown to have antihypertensive activity. However, the role of epigallocatechin gallate (EGCG) in improving vascular function via modulation of endothelial nitric oxide synthase (eNOS) in hypertensive subjects is not well researched. Angiotensin II-infused hypertensive mice (8–10 weeks old) received EGCG (50 mg/kg/day) for 14 days via oral gavage. The arterial systolic blood pressure (SBP) was measured using the tail-cuff method every three days. At the end of the treatment, the vascular reactivity of the isolated aortae was studied using wire myographs. The level of nitric oxide (NO), cyclic guanosine monophosphate (cGMP) and tetrahydrobiopterine (BH_4_) were determined using assay kits while the presence of proteins (NOS, p-eNOS and NOx-2) were determined using by Western blotting. In vivo treatment with EGCG for 14 days significantly attenuated the increase in SBP, alleviated the vascular dysfunction, increased the vascular cGMP and BH_4_ level as well as the expression of p-eNOS and decreased elevated ROS level and NOx-2 protein in angiotensin II-infused hypertensive mice. Collectively, treatment with EGCG in hypertensive mice exerts a blood pressure lowering effect which is partly attributed to the improvement in the vascular function due to its ability to reduce vascular oxidative stress in the aortic tissue leading to a decrease in eNOS uncoupling thus increasing NO bioavailability.

## Introduction

Hypertension or elevated blood pressure is a highly prevalent medical condition that significantly increase the risk of cardiovascular diseases such as coronary heart disease, heart failure, stroke, myocardial infarction and stroke^1,2^. Hypertension is estimated to affect up to 1.4 billion adults worldwide, mostly those who live in low- and middle-income countries^3^. A common phenomenon observed among hypertensive patients is an increase in total peripheral resistance with a normal cardiac output^4^. One of the factors that attributes to the increase in peripheral resistance is the abnormalities in the structure and function of the vasculature leading to the occurrence of endothelial dysfunction^5^.

Endothelial dysfunction is characterized by a decreased release of endothelium-derived relaxing factors such as nitric oxide (NO), prostacyclin (PGI_2_) and endothelium-derived hyperpolarizing factor (EDHF) accompanied by an enhanced production of endothelium-derived contracting factors such as thromboxane A_2_ (TxA_2_), superoxide (O_2_^−^) anions, endothelin-1 (ET-1), hydrogen peroxide (H_2_O_2_) and angiotensin II^5^. This leads to an impairment in endothelium-dependent relaxation due to a compromised nitric oxide (NO)-cyclic GMP (cGMP) signaling pathway which later mediates vasodilation^6^.

NO has been associated with the physiological and pharmacological function in various vascular vessels including the control of pulmonary and systemic vascular tone, cardiac and vascular remodeling, platelet aggregation and vascular smooth muscle cell proliferation^7^. The production of NO from endothelial nitric oxide synthase (eNOS) is the major source of endothelium-derived NO and thus plays an important role in maintaining the relaxation and contraction of vasculature^8^. The generation of NO from its precursor L-arginine by eNOS requires the presence of several cofactors such as nicotinamide adenine dinucleotide phosphate (NADPH), tetrahydrobiopterin (BH_4_) and calcium-calmodulin. The monomers of eNOS form dimers and bind to BH_4_ and L-arginine in order to produce NO^9^. BH_4_ facilitates the transferring of NADPH-derived electron from the eNOS reductase to the oxygen domain for the conversion of L-arginine to NO and L-citrulline^10^. In the presence of oxidative stress or a decrease in BH_4_ level, the coupled form of eNOS will be disrupted and becomes uncoupled^11^. eNOS uncoupling has been demonstrated to be the underlying cause of endothelial dysfunction in experimental animals such as atherosclerosis rat^12^, transit middle cerebral artery occlusion (tMCAO) mice^13^, as well as in spontaneously hypertensive rats^14^. The uncoupling of eNOS is linked to the enhanced oxidation of BH_4_ by reactive oxygen species (ROS) which is derived from vasculature NADPH oxidases. It is associated with a decrease in the bioavailability of NO and further worsen endothelial dysfunction^15^.

Treatments that aim to address endothelial dysfunction have been a major focus in preventing complication in patients since endothelial dysfunction is associated with cardiovascular diseases. In recent years, there has been a growing interest in the usage of tea, which is a popular drink in Asian countries, as an alternative management of cardiovascular diseases. Among the types of teas, the demand on green tea has been increasing due to its fine taste and beneficial health effects^16^. Green tea is a type of tea that is made from *Camellia sinensis* leaves. It contains a wide variety of polyphenols compounds such as flavanols, flavandiols, flavonoids, and phenolic acids which accounts to 30% of its dry weight. Flavanols or commonly known as catechins is one of the most abundant green tea polyphenols. Among the catechins found in green tea, epigallocatechin gallate (EGCG) is predominant and is the most abundant polyphenol accounting for as much as 50% of green tea polyphenols^17^.

EGCG has been demonstrated to possess strong antioxidative and anti-inflammatory properties which are beneficial to the cardiovascular health^18^. Study done by Alvarez-Cilleros and colleagues has demonstrated that EGCG prevented endothelial dysfunction in human umbilical vein endothelial cells (HUVECs) by decreasing the generation of ROS and the activation of stress-related pathway^19^. In line with this, in vivo study done by Qian and colleagues has also showed that green tea administration in spontaneously hypertensive rat (SHR) helped to control development of hypertension, which is benefited from the ACE-inhibitory and antioxidative properties of catechins^20^. EGCG has also been shown to inhibit NADPH oxidase subunits, p47^phox^ and p22^phox^ in the vascular endothelial cells of SHR^21,22^. Furthermore, studies have been demonstrated that EGCG leads to endothelium-dependent vasorelaxation in rat thoracic aorta^23,24^.

Although studies have shown that EGCG has endothelium protective effects as well as blood lowering effects, there is still a lack of evidence to link the roles of EGCG in the modulation of eNOS and vascular function to is hypotensive effect. Therefore, this study investigated the modulation of eNOS by EGCG to improve endothelial dysfunction in angiotensin II-infused hypertensive model, an experimental model which exhibits a dysfunctional endothelium due to augmented production of NADPH-derived ROS^25,26^.

## Materials and methods

### Animals preparation and treatment

Male C57BL/6J mice (8–10 weeks old) were purchased from Monash University (Sunway Campus, Malaysia). All animal studies were in compliance with the ARRIVE guidelines for experiments involving animals which was agreed and approved by the Ethics Committee of Universiti Tunku Abdul Rahman (U/SERC/88/2018) and University of Malaya (2018-210807/PHAR/R/DDM). Mice were maintained at a controlled temperature (24 ± 1 °C) and lighting condition (12-h light/dark cycle) with free access to standard chow and water.

### Establishment of angiotensin II-induced hypertensive animal model and treatment

Angiotensin II (1.2 mg/kg/day) or phosphate buffer saline (PBS) were loaded into Alzet osmotic minipumps (Model 1002; DURECT Corporation, Cupertino, CA, USA). The pumps were implanted subcutaneously into the mice under ketamine plus xylazine anesthesia (75 mg/kg and 6 mg/kg body weight, respectively; intraperitoneal injection) and was left in the mice for 2 weeks^27^. The mice were then randomly divided into four experimental groups: (1) control, (2) control + epigallocatechin gallate (EGCG) treated, (3) angiotensin II control and (4) angiotensin II + EGCG treated. The dosage of EGCG used is 50 mg/kg body weight^28,29^ and is administered via oral gavage. The treatment was given to the animals on the next day after the osmotic pump was inserted. The animals were treated for 14 days before they were sacrificed.

### Measurement of systolic blood pressure (SBP)

The average systolic blood pressure (SBP) of the animals was measured non-invasively by the tail-cuff method (CODA Surgical Monitor, United States), before all treatments were initiated and every three days thereafter. This method uses a specialized volume pressure recording (VPR) sensor and measures the blood volume changes that are placed over the animal's tail. All animals were trained before the actual measurement was done to ensure the true blood pressure of the animal was obtained. Training prior to the actual measurement of blood pressure enables the mice to adapt to the procedures and hence ensure a more reproducible result. During measurement of blood pressure, the animals were immobilized in a pre-warmed chamber at around 28–30 °C for at least 15 min before the readings was taken. At least five to six successive measurements were recorded and the average values of all readings were computed and reported.

### Collection of samples

At the end of the treatment period, all mice were euthanized via excess carbon dioxide inhalation. Blood samples from the animals were collected immediately through cardiac puncture. The thoracic aortae were isolated and placed in ice-cold Krebs solution (control solution, in mM: NaCl 118.93, NaHCO_3_ 25.00, MgSO_4_ 1.18, KCl 4.69, KH_2_PO_4_ 1.03, Glucose 11.10, and CaCl_2_ 2.38) and the adhering connective tissues and fat were trimmed off. The cleaned aorta was cut into segments of 2–4 mm in length for vascular reactivity study. A portion of the aortic tissue segments were placed in OCT compound or snap frozen using liquid nitrogen and stored in − 80 °C for later processing.

### Measurement of vascular tissue reactivity

For the measurement of isometric tension, the rings were suspended in a myograph (Danish Myo Technology, Aarhus, Denmark) containing 5 mL control solution (37 °C), continuously aerated with 95% O_2_ and 5% CO_2_ throughout the experiment. The changes in isometric tension were recorded using a Powerlab recording system (AD Instruments, Sydney, Australia). After an equilibrium period of 30 min at their optimal resting tension (3mN), the rings’ viability was tested by a contraction-response test with 60 mM KCl until a stable contraction was achieved. After that, the rings were washed three times with the control solution. The endothelial function of the aortic rings was investigated by cumulative addition of acetylcholine (ACh; 3 nM–10 µM) to phenylephrine (PE; 300 nM–1 µM)-contracted rings to induce endothelium-dependent relaxation. Cumulative concentration–response to sodium nitroprusside (SNP; 1 nM–10 µM) were also obtained to investigate if there are any changes of the vascular smooth muscle sensitivity to NO.

### Measurement of plasma nitrite levels

The collected blood samples collected was centrifuged at 2000 g at 4 °C for 10 min and the supernatant was collected. Around 100 µL of plasma from each sample was used for the determination of the plasma nitrite levels. The assay was performed using a commercially available colorimetric assay kit (Cayman Chemical Company, Ann Arbor, MI, USA). Briefly, the 100 µL plasma was added into the wells of a 96-wells plate accordingly. Then, 50 µL of Griess Reagent 1 and 2 were added into each well. The color was allowed to develop for 10 min at room temperature. The absorbance was measured using a plate reader (Tecan, Männedorf, Switzerland) with an absorbance at 540 nm. The results were expressed in μM.

### Measurement of vascular nitric oxide (NO) levels

4-Amino-5-Methylamino-2′,7′-Difluorofluorescein (DAF-FM) diacetate reagent was used to detect and quantify low concentrations of vascular nitric oxide (NO). Briefly, isolated aortae were embedded in OCT compound (Sakura Finetik, AJ Alphen aan den Rijn, Netherlands) and kept frozen. The frozen segments were cut into cryostat sections of 10 μm thick. The cut sections were then incubated with 5 μM DAF-FM diacetate (Invitrogen, Carlsbad, CA, USA) for 15 min in normal physiological saline solution [NPSS (mM): NaCl 140, KCl 5, CaCl_2_ 1, MgCl_2_ 1, glucose 10 and HEPES 5] at 37 °C. The fluorescence dye was then washed and the sections were visualized using a fluorescence microscope (Zeiss Axio Observer A1) with excitation at 495 nm and emission at 515 nm. The fluorescence intensity was measured using the Zen Lite software version 2.3. The NO level was compared to that of the control.

### Measurement of vascular cyclic guanosine monophosphate (cGMP) levels

Frozen aortic tissue samples were used for the measurement of total cGMP level. Prior to the assay, the aortic tissues were homogenized in 5% tricloroacetic acid (TCA) and centrifuge at 15,000 g for 15 min at room temperature. The supernatant obtained was used for the measurement of total cGMP levels. The assay was performed by using available commercial assay kit (Cayman Chemical Company, Ann Arbor, MI, USA). The absorbance was measured using a plate reader (Tecan, Männedorf, Switzerland) at 405 nm. The results were expressed in pmol/mg protein.

### Measurement of vascular tetrahydrobioterin (BH_4_) levels

Frozen aortic tissue samples were used for the measurement of total BH_4_ level. Prior to the assay, the aortic tissues were homogenized in 5% tricloroacetic acid (TCA) and centrifuged at 15,000 g for 15 min at room temperature. The supernatant obtained was used for the measurement of total BH_4_ levels. The assay was performed by using available commercial assay kit (Novus Biologicals, USA). The absorbance was measured using a plate reader (Tecan, Männedorf, Switzerland) at 405 nm. The results were expressed in pmol/mg protein.

### Detection of vascular reactive oxygen species (ROS) formation

Dihydroethidium (DHE)-mediated fluorescence microscopy was used to determine the intracellular ROS production at the vascular tissues. Briefly, isolated aortae were embedded in OCT compound (Sakura Finetik, AJ Alphen aan den Rijn, Netherlands) and frozen. The frozen segments were cut into cryostat sections of 10 μm thick. The cut sections were then incubated in dark for 15 min with 5 μM DHE (Invitrogen, Carlsbad, CA, USA) in normal physiological saline solution [NPSS (mM): NaCl 140, KCl 5, CaCl_2_ 1, MgCl_2_ 1, glucose 10 and HEPES 5] at 37 °C. The fluorescence dye was then washed with PBS and the sections were visualized using a fluorescence microscope (Zeiss Axio Observer A1) with excitation at 495 nm and emission at 515 nm to visualize the signal for ROS. The fluorescence intensity was measured using the Zen Lite software version 2.3. The ROS level was compared to that of the control.

### Western blotting

Frozen sections of isolated aorta were homogenized in ice-cold RIPA buffer (leupeptin 1 μg/mL, aprotonin 5 μg/mL, PMSF 100 μg/mL, sodium orthovanadate 1 mM, EGTA 1 mM, EDTA 1 mM, NaF 1 mM, and β-glycerolphosphate 2 mg/mL). The lysate was centrifuged at 15,000 g (30 min, 4 °C) and the supernatant was used for Western blotting. A modified Lowry assay (Bio-Rad Laboratories, Hercules, CA, USA) was used to determine the protein concentrations. A total protein of ten microgram per sample were separated on a 10% sodium dodecyl sulphate (SDS)-polyacrylamide gel and transferred to an immobilon-P polyvinylidene difluoride membrane (Millipore, Billerica, MA, USA) at 110 V for 90 to 120 min. The blots were then blocked by incubation with 3% bovine serum albumin (BSA) in Tris buffered saline containing 0.2% Tween-20 (TBS-T) with gentle shaking at room temperature. Later, the blots were incubated overnight with primary antibodies against phosphorylated eNOS (peNOS 1:500, Abcam), eNOS (1:1000, Abcam), NOX-2 (1: 1000, Abcam) and β-actin (1:10,000, Sigma-Aldrich) at 4 °C. The membranes were washed with TBS-T for three times the next day, followed by the incubation with respective HRP-conjugated secondary antibodies at room temperature for two hours. After that, the blots were developed with enhanced chemiluminescence detection system and images were captured for quantification of protein abundance. p-eNOS protein expression level was normalized to eNOS while Nox-2 protein expression was normalized to housekeeping protein, β-actin and compared with control-vehicle.

### Drugs and chemicals

Phenylephrine, acetylcholine chloride, sodium nitrite, sodium nitroprusside, and Tween-20 were purchased from Sigma Chemicals (St Louis, MO, USA). Angiotensin II was purchased from Sigma Chemicals (USA). EGCG was purchased from Cayman. NaCl was purchased from Calbiochem® Merck (Darmstadt, Germany). MgSO_4_, KCl, KH_2_PO_2_, glucose and CaCl_2_ were purchased from BDH Laboratory Supplies (Poole, UK). Bovine serum albumin (BSA) was purchased from Santa Cruz (Dallas, Texas, USA). All compounds were dissolved in deionized water. The concentrations stated are expressed as final molar concentrations in the buffer.

### Data analysis

The results are presented as means ± standard error of mean (SEM) for the number of mice (n) in each group. GraphPad Prism 6 (GraphPad Software, La Jolla, CA, USA) was used to analyze the concentration response curves by non-linear regression fitting. Student's unpaired t test (to compare and evaluate the differences between individual groups) and one-way ANOVA followed by Benferroni’s multiple comparison tests (to compare and evaluate the differences between multiple groups) were performed using the same statistical software. p values of less than 0.05 was considered statistically significant.

### Institutional Review Board statement

The study was conducted in compliance with the Malaysian Code of Practice for The Care and Use of Animals for Scientific Purposes for experiments involving animals which was agreed and approved by the Ethics Committee of Universiti Tunku Abdul Rahman (U/SERC/88/2018) and University of Malaya (2018-210807/PHAR/R/DDM).

## Results

### Effect of EGCG on systolic blood pressure (SBP) of angiotensin II-infused hypertensive mice

**Figure 1:**
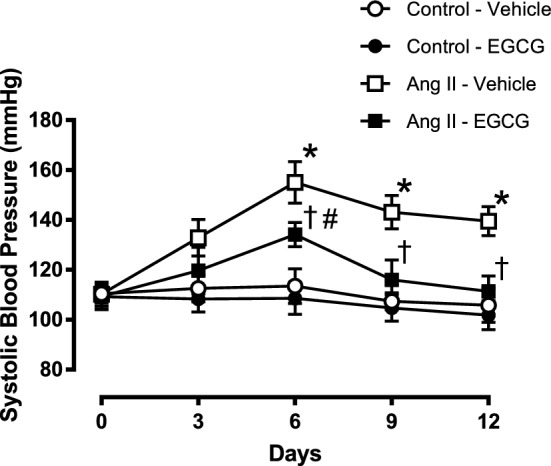
The average systolic blood pressure (SBP) in control and angiotensin (Ang) II-infused hypertensive mice with and without in vivo treatment with epigallocatechin gallate (EGCG). Data are expressed as means ± SEM (n = 6–7). *p ≤ 0.05 Ang II—vehicle compared to control—vehicle; ^†^p ≤ 0.05 Ang II—vehicle compared to Ang II—vehicle; ^#^p ≤ 0.05 Ang II—vehicle compared to control—vehicle.

The basal systolic blood pressure of all animals was similar (Fig. 1). Infusion with angiotensin II increased the systolic blood pressure of the animals starting on the second day. This increase was continuous in angiotensin II-infused animals without EGCG (50 mg/kg) treatment. There is an increase in SBP of angiotensin II-infused hypertensive mice treated with EGCG but this increase was attenuated after 6 days of treatment. At the end of the experiment, the SBP of angiotensin II-infused mice treated with EGCG was significantly lower by 40% compared to that of angiotensin II-infused hypertensive mice without treatment. There were no significant changes observed in the SBP of control C57BL/6 J mice with and without EGCG treatment.

### Effect of EGCG on vascular reactivity in the aorta of angiotensin II-infused hypertensive mice

**Figure 2:**
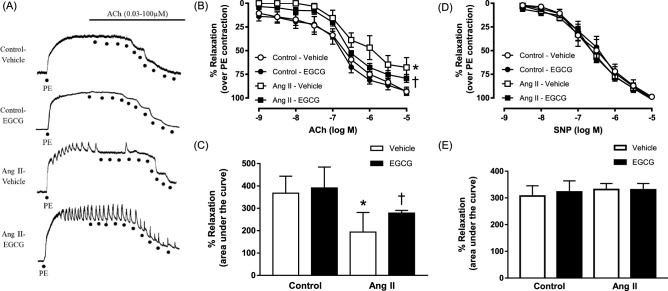
Relaxation to increasing concentration of acetylcholine (ACh) and sodium nitroprusside (SNP) in aortic rings of control and angiotensin (Ang) II-infused hypertensive C57BL/6J mice. (**A**) Representative traces of acetylcholine-induced relaxation in phenylephrine (PE)-contracted aortic rings of all experimental groups. (**B**) Relaxation curves and (**C**) area under the curve for percentage of relaxation in aortic rings of control and angiotensin II-infused hypertensive mice, with and without in vivo treatment with epigallocatechin gallate (EGCG) to ACh. (**D**) Relaxation curves and (**E**) area under the curve to SNP of all experimental groups. Data are expressed as means ± SEM (n = 5–6). *p ≤ 0.05 compared to control—vehicle; ^†^p ≤ 0.05 compared to Ang II—vehicle.

In the study of vascular reactivity, 2 weeks of angiotensin II infusion resulted in a 30% decrease in relaxation to acetylcholine in aortic rings of angiotensin II-infused hypertensive mice compared to control mice (Fig. 2A–C). Co-treatment with EGCG in angiotensin II-infused hypertensive mice showed a significant improvement in acetylcholine-induced relaxation compared to that of non-treated angiotensin II-infused hypertensive mice. The relaxation to acetylcholine was unaltered in control mice under similar treatment with EGCG. No significant changes in relaxation were observed in aortae of control mice either with or without EGCG treatment. The relaxations to sodium nitroprusside, an exogenous NO donor were comparable in all experimental groups (Fig. 2D and E).

### Effect of EGCG on total plasma nitrate and nitrite and vascular NO levels in angiotensin II-infused hypertensive mice

**Figure 3:**
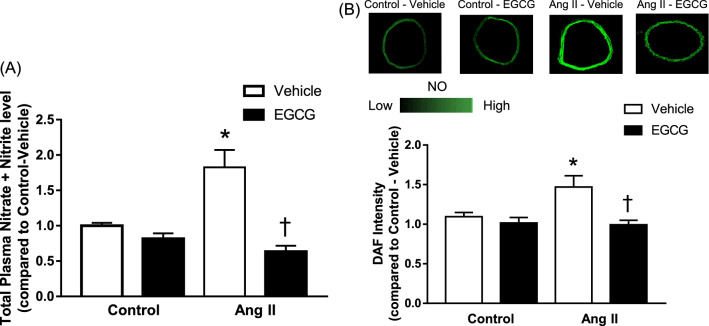
Plasma nitrite level (**A**) and vascular NO level (**B**) of control and angiotensin (Ang) II-infused hypertensive C57BL/6J mice, with or without in vivo treatment with epigallocatechin gallate (EGCG) measured using commercially available kit and DAF-FM staining, respectively. The upper panel shows representative fluorescence images of the stained aortae and the bottom panel shows the quantified fluorescence intensity (**B**). Data are expressed as means Data are expressed as means ± SEM (n = 5–6). *p ≤ 0.05 compared to control—vehicle; ^†^p ≤ 0.05 compared to Ang II—vehicle.

The measurement of total plasma nitrate and nitrite levels shows that mice infused with angiotensin II exhibited a two-fold increase in total plasma nitrate and nitrite compared to that of control animals (Fig. 3A). 2 weeks of in vivo treatment with EGCG significantly decreased the level of total plasma nitrate and nitrite in angiotensin II-infused hypertensive mice. No significant changes were observed in either treated or non-treated control group.

In situ measurement of NO production by DAF-FM diacetate fluorescence staining demonstrated that the basal NO level in aortic rings of angiotensin II-infused hypertensive mice is signficantly higher compared to that of the control group (Fig. 3B). After 2 weeks of treatment with EGCG, there was a significant decrease in the fluorescence intensity in angiotensin II-infused hypertensive mice aortic rings, implying that there is a lower level of NO in the aortic tissue. No significant changes were observed in either treated or non-treated control group.

### Effect of EGCG treatment on vascular BH_4_, cGMP and phosphorylated eNOS levels in angiotensin II- infused hypertensive mice

**Figure 4:**
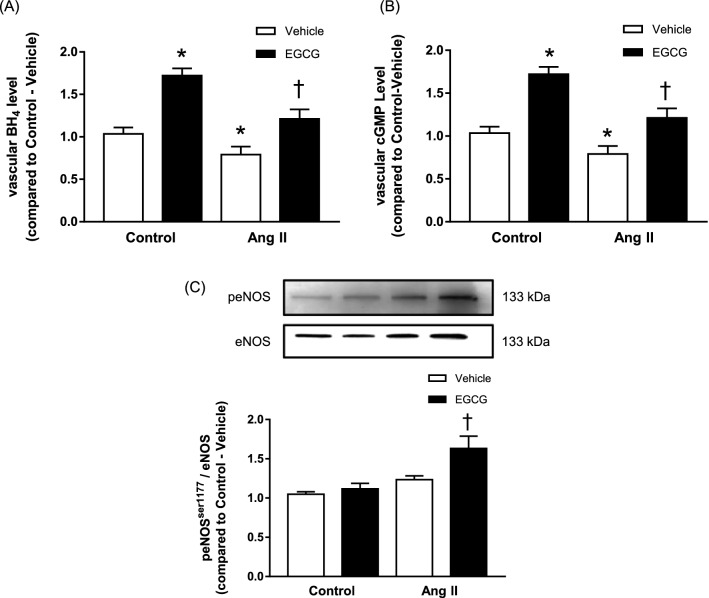
Vascular BH_4_ (**A**) and vascular cGMP (**B**) levels measured using commercially available ELISA kits and the presence of total phosphorylated eNOS (p-eNOS) at ser^1177^ (**C**) detected by Western blotting in the aortic tissues of control and angiotensin (Ang) II-infused hypertensive C57BL/6J mice, with and without in vivo treatment with epigallocatechin gallate (EGCG). The upper panel shows representative Western blots and the bottom panel shows the ratio of protein to eNOS (**C**). Data are expressed as means ± SEM (n = 5–6). *p ≤ 0.05 compared to control—vehicle; ^†^p ≤ 0.05 compared to Ang II—vehicle.

Mice infused with angiotensin II treatment exhibited a significantly lower level of BH_4_ compared to the control group (Fig. 4A). In vivo treatment with EGCG for 2 weeks significantly increased the level of BH_4_ in mice infused with angiotensin II. Similarly, there was also a significant increase in the BH_4_ level following treatment with EGCG in the control group.

The measurement of vascular cGMP level demonstrated that angiotensin II-infused hypertensive mice exhibited a significantly lower level of cGMP compared to the control group (Fig. 4B). In vivo treatment with EGCG for 2 weeks significantly increased the level of cGMP in angiotensin II-infused hypertensive mice. There was also a significant increase in the cGMP level following treatment with EGCG in the control group.

The Western blot result demonstrated that the total phosphorylated eNOS (p-eNOS) protein level in the aortic tissue of angiotensin II control group is similar to that of the normotensive control group with and without treatment with EGCG (Fig. 4C). 2 weeks treatment with EGCG significantly increased the level of p-eNOS in both control and angiotensin II-infused hypertensive mice group. Treatment with EGCG increased the aortic tissue protein level of phosphorylated eNOS level in angiotensin-II infused hypertensive mice to a significant level but did not influence the phosphorylated eNOS level in the aortae of control mice receiving the same treatment (Supplementary information).

### Effect of EGCG treatment on vascular ROS level and NOx-2 protein expression in mice infused with angiotensin II

**Figure 5:**
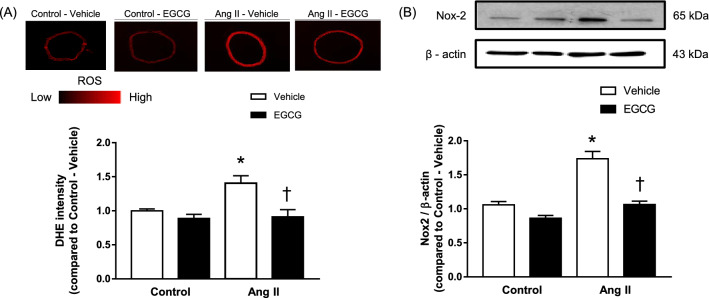
Vascular ROS level (**A**) and the presence of Nox-2 protein (**B**) in aortic tissue of control and angiotensin (Ang) II-infused hypertensive C57BL/6J mice with and without in vivo treatment with epigallocatechin gallate (EGCG) measured by DHE fluorescence. The upper panel shows representative fluorescence images of the stained aortae and the bottom panel shows the quantified fluorescence intensity (**A**). The upper panel shows representative Western blots and the bottom panel shows the ratio of protein to β-actin (**B**). Data are expressed as means ± SEM (n = 5–6). *p ≤ 0.05 compared to control–vehicle; ^†^p ≤ 0.05 compared to Ang II—vehicle.

Measurement of in situ ROS level by DHE florescence staining revealed that aorta tissue of angiotensin II-infused hypertensive mice has significantly higher level of ROS compared to the control group (Fig. 5A). 2 weeks of in vivo treatment with EGCG attenuated the ROS level in mice infused with angiotensin II to a level that is comparable with the control groups. Treatment with EGCG for 2 weeks has no effect on the ROS level between the treated and non-treated with EGCG in control mice.

In line with that, the measurement of Nox-2 protein in angiotensin II-infused hypertensive mice aortic rings exhibited a higher level of Nox-2 protein compared to the control group (Fig. 5B). 2 weeks of in vivo treatment with EGCG significantly decreased the expression of Nox-2 level in angiotensin II-infused hypertensive mice aortae. There were no significant changes in the protein expression of Nox-2 between treated and untreated with EGCG in control animals (Supplementary information).

## Discussion

The present study demonstrated that treatment with EGCG attenuated the increase in arterial SBP caused by angiotensin II-infusion. The decreased in SBP is accompanied by an improvement in acetylcholine-induced endothelium-dependent relaxation in the aortae. Furthermore, an increase in BH_4_ and cGMP levels as well as and the expression of p-eNOS was observed in the aortic tissues of angiotensin II-infused hypertensive mice treated with EGCG. Adding on, treatment with EGCG has also resulted in a decrease in ROS level and the presence of Nox-2 protein in the aorta of angiotensin II-infused hypertensive mice. These findings suggest that the blood pressure lowering effect of EGCG which is associated with an improvement in the endothelial function, may be partly attributed by its ability to reduce oxidative stress and eNOS uncoupling thus increasing NO bioavailability.

The infusion of angiotensin II for 14 days resulted in an elevation in the SBP of C57BL/6J starting on the second day of infusion which is in line with previous studies^30,31^. Angiotensin II-infused mice that received EGCG treatment starting on the second day of angiotensin II infusion is observed to have an attenuation in the increase of their SBP. EGCG is one of the most abundant polyphenols that is found in green tea. The observation whereby EGCG prevented the elevation of SBP in hypertensive animal is in line with previous studies involving hypertensive subjects. Treatment with EGCG has been demonstrated to decrease the SBP, reduce proteinuria and ameliorate renal fibrosis in Dahl rats with salt-sensitive hypertension^32^. Pre-treatment with EGCG has also significantly attenuated the inhibition of vasodilator response to acetylcholine induced by native low density lipoprotein in Sprague–Dawley rats^33^. On the other hand, a study done by Bogdanski and colleagues has demonstrated that a three month-supplementation of green tea extract (GTE) significantly decreased the systolic and diastolic blood pressure in obese, hypertensive subjects^34^. In line with this, a meta-analysis of 14 random clinical trials (RCTs) also demonstrated that GTE supplementation caused a small but significant reduction in the blood pressure among overweight and obese adults^35^. Similarly, favourable effects on both systolic and diastolic blood pressure were also observed in pre-hypertensive and hypertensive individuals after consuming green tea for two months^36^.

An elevation in the level of angiotensin II upregulates the vascular NADPH oxidase leading to excessive production of superoxide anions which results in a decrease in the bioavailability of NO and thus the occurrence of endothelial dysfunction^37,38^. This is in line with the current observation whereby the mice receiving angiotensin II infusion exhibited a decrease in relaxation to acetylcholine after 14 days of infusion. Since hypertension is highly associated with endothelial dysfunction^39^, a decrease in blood pressure may be accompanied by an improvement in endothelial function. This is evident from the present study whereby treatment with EGCG for 14 days resulted in an improvement in the endothelium-dependent relaxations to acetylcholine in the aorta of angiotensin II-infused hypertensive mice. The present result is in agreement with the study done by Potenza and colleagues which demonstrated that treatment with EGCG for 3 weeks in SHR resulted in a significant improvement in insulin-induced vasodilation and a significant decrease in blood pressure of the treated animals^21^. The decreased in systolic blood pressure of angiotensin II-infused hypertensive mice treated with EGCG accompanied by an improvement in endothelium-dependent suggests that the antihypertensive effect of EGCG may be in part attributed to the improvement in endothelial-dependent relaxation of the animals receiving EGCG treatment. It is also worthy to note that the beneficial effect of in vivo treatment with EGCG is not due to an enhanced sensitivity of the murine vascular smooth muscle to NO, as the relaxations to sodium nitroprusside, an exogenous NO donor, was unaltered in all treatment group.

The increased spontaneous contractions to acetylcholine in the aortas of angiotensin II-infused mice treated with EGCG could be explained by the release of PGI_2_ in response to acetylcholine. PGI_2_ has been demonstrated to evoke contractions in various vascular preparations including human coronary arteries^40–42^. It has been demonstrated that acetylcholine-induced endothelium-dependent contraction and endothelium-dependent release of PGI_2_ were superimposable for hypertensive or non-hypertensive animals. The rapid time-course of PGI_2_ release is also compatible with the time course of endothelium-depended contractions. In addition, exogenous PGI_2_ has been demonstrated to elicit endothelium-independent contraction that mimicked the endothelium-dependent contractions elicited by acetylcholine. These contractions were in both cases transient of small magnitude and virtually abolished by the presence of a functional NO-synthase. Therefore, the increased spontaneous contractions to acetylcholine could be at least in part or possibly entirely by the release of PGI_2_^43,44^.

NO acts as a paracrine regulator of the underlying vascular smooth muscle tone by activating sGC to produce cGMP which then induces the relaxation of the vascular smooth muscle^45^. As EGCG treatment improved impaired endothelial function in the present study, it was hypothesized that treatment with EGCG may increase the plasma NO level which contributes to the observed increase in endothelium-dependent relaxation to acetylcholine thus leading to the observed decrease in blood pressure. However, contrasting result was observed. In the present study, an increased level of total plasma nitrate and nitrite and DAF-FM diacetate fluorescence intensity was observed from the aortic rings of mice infused with angiotensin II compared to that of control animals. In vivo treatment with EGCG for 2 weeks significantly decreased the level of total plasma nitrate and nitrite as well as the vascular NO level in both control and angiotensin II-infused hypertensive mice. The increase in NO level in angiotensin II-infused hypertensive animals after 2 weeks was in line with the study done by Ling and colleagues^27^. The group has demonstrated that the level of plasma nitrate increased significantly in angiotensin II-infused hypertensive mice after 2 weeks. The increase in NO level in angiotensin II-infused hypertensive mice was suggested to be due to the production of NO from iNOS^27,46,47^. Activation of iNOS generates large amounts of undesired NO and contributes to the pathophysiology of inflammatory diseases and septic shock. The excessive production of NO by iNOS interacts with superoxide anions (O_2_^−•^) and lead to the formation of peroxynitrite (ONOO^−^), a more toxic radical that contributes to endothelial dysfunction in hypertension^5^. Indeed, increase in iNOS expression has been previously demonstrated in Dahl salt-sensitive model of HFpEF and SHR aorta that exhibits several common characteristics with human hypertension^48,49^. In line with that, the decrease in total plasma nitrate and nitrite level and vascular NO level after 2 weeks of treatment with EGCG in this study may suggests that there is a possibility of attenuation of iNOS activity. Further study involving the measurement of the level of iNOS will indeed help to shed light if treatment with EGCG attenuates the vascular iNOS level in angiotensin II-infused hypertensive mice.

NO synthesis by eNOS can be reduced by several factors which include interaction with endogenous modulators or suboptimal concentrations of substrate and cofactors, post translational modifications and changes in mRNA expression which resulting in decreased protein presence^50^. The present study has demonstrated that treatment with EGCG for 2 weeks increased the expression of phosphorylated eNOS in angiotensin II-infused hypertensive mice, suggesting that EGCG is involved in activation of eNOS, which eventually leads to the production of NO. Endothelium-derived NO after diffusion into the underlying smooth muscle cells, binds to the heme moiety of sGC, leading to the production of cGMP, hence activation of cGMP-dependent protein kinases, and ultimately causes relaxation^7^. The present findings demonstrate that the aortae of angiotensin II-infused hypertensive mice treated with EGCG exhibited increased in cGMP levels compared to control animals, providing further support that the beneficial effect of EGCG may be due to at least in part to the upstream activation of eNOS followed by increased production of NO. This study is in line with previous study whereby treatment with EGCG induced a rate-determining process of 67LR-dependent cell death through upregulation of cGMP^51^.

BH_4_ is the key regulator of eNOS in the setting of cardiovascular health and disease^52^. It is a critical determinant of NO synthesis and is crucial for the maintenance of normal endothelial function^53^. Previous studies have shown that in the state of vascular disease, there is an increase in oxidative degradation of BH_4_ by reactive oxygen species^54,55^. When the BH_4_ level is limiting, electron transfer from NADPH through the flavins to molecular oxygen is not inhibited thus eNOS can no longer produce NO but instead will generate superoxide which further aggravates endothelial dysfunction^53^. In line with this, the current study demonstrated that there is a decreased BH_4_ level and an increase in DHE fluorescence intensity in aortic tissue of angiotensin II-infused hypertensive mice which is accompanied by an increase in vascular NOx-2 level. NADPH oxidase has been implicated as an important pro-oxidant enzyme which can contribute to hypertension^56^. The increase in NOx-2 level may lead to an increase in ROS level and subsequently led to a degradation in aortic BH_4_ level. The increase in BH_4_ level and a decrease in DHE fluorescence intensity as well as the level of NOx-2 following treatment with EGCG for 2 weeks demonstrates that the EGCG may increase the bioavailability of NO by increasing eNOS activity via activation of BH_4_. In situation whereby the NO level is low, BH_4_-eNOS uncoupling will occur and lead to an increase in the production of vascular O_2_^–^^54^. This subsequently leads to impaired vascular relaxation which worsen the peripheral vascular resistance and led to an increase in blood pressure, a condition which will develop into hypertension if leave untreated. Therefore, the ability of BH_4_ to modulate both NO production and superoxide production in the endothelium is an important mechanism underlying endothelial dysfunction to treat vascular disease.

In a summary, the findings from this study suggest that the antihypertensive effect of EGCG may be attributed, at least in part, to its ability to reduce oxidative stress through downregulation of vascular NADPH oxidase activity, leading to a decrease in BH_4_-eNOS uncoupling thus increasing NO bioavailability, leading to improvement in endothelial function in hypertensive animals. Thus, EGCG may be further explored to be used as a complementary treatment for hypertension and as a supplementation for the patients with cardiovascular diseases.

## Supplementary Information

Supplementary Information.

## Data Availability

The datasets used and/or analysed during the current study available from the corresponding author on reasonable request.
