# Supplementary material for: Epigallocatechin gallate (EGCG) alleviates vascular dysfunction in angiotensin II-infused hypertensive mice by modulating oxidative stress and eNOS
Source: Sci Rep. 2022 Oct 21;12:17633. doi: 10.1038/s41598-022-21107-5 (PMC9587239; doi:10.1038/s41598-022-21107-5)

Western blot representative image_p-eNOS

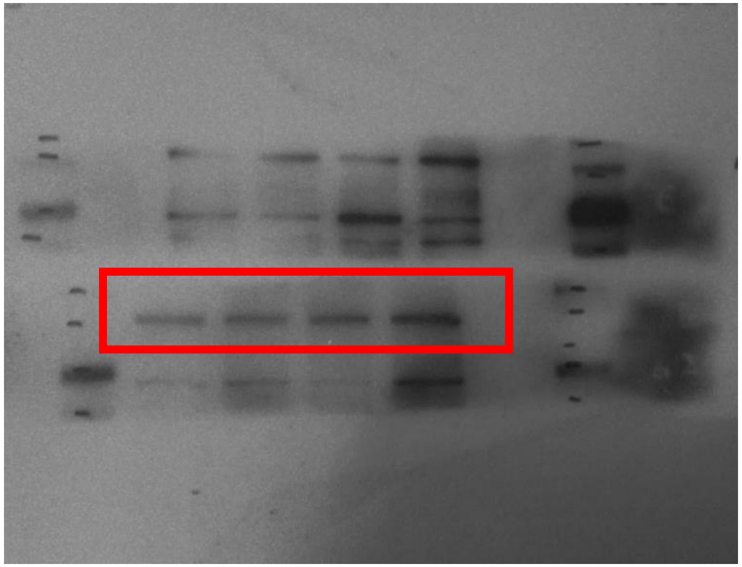

Western blot representative image_eNOS

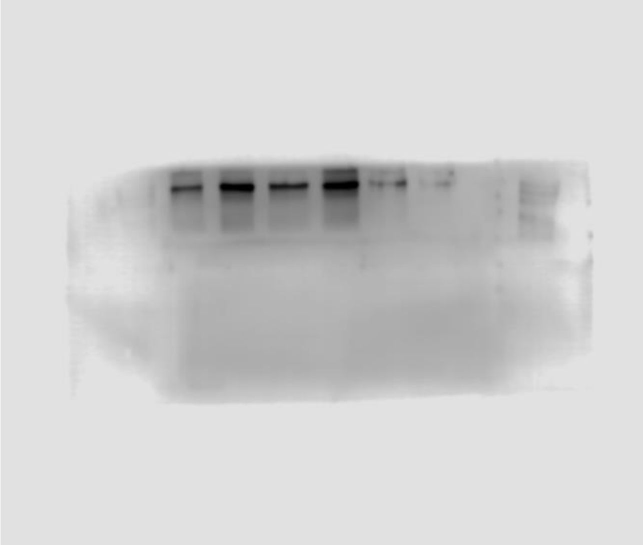

Western blot representative image_Nox-4

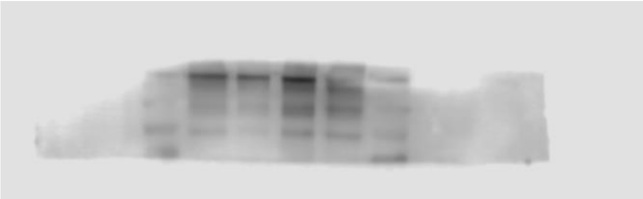

Western blot representative image_β-actin

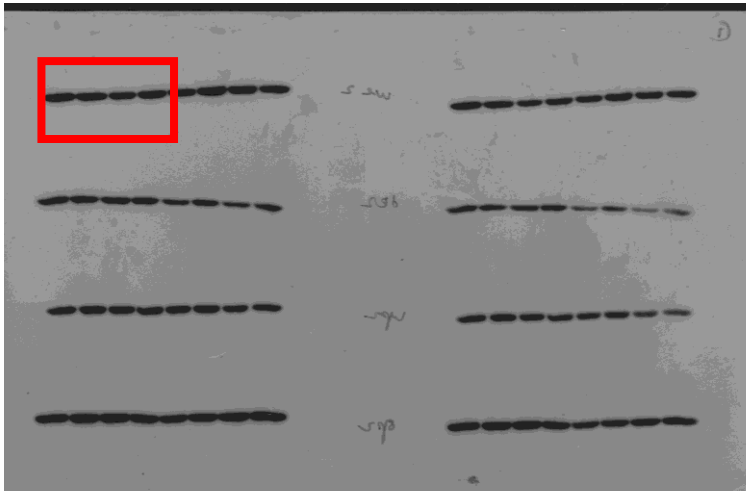

Supplement: Supplementary file 1 — Supplementary Information. [file 41598_2022_21107_MOESM1_ESM.pdf]
